# Intratumor Hypoxia Promotes Immune Tolerance by Inducing Regulatory T Cells via TGF-β1 in Gastric Cancer

**DOI:** 10.1371/journal.pone.0063777

**Published:** 2013-05-27

**Authors:** Bin Deng, Ji-Min Zhu, Yi Wang, Tao-Tao Liu, Yan-Bing Ding, Wei-Ming Xiao, Guo-Tao Lu, Ping Bo, Xi-Zhong Shen

**Affiliations:** 1 Department of Gastroenterology, Second Clinical School of Yangzhou University, Yangzhou, Jiangsu, China; 2 Department of Gastroenterology, Zhongshan Hospital of Fudan University, Shanghai, China; 3 Institute of Integrated Chinese Traditional and Western Medicine, Medical College of Yangzhou University, Yangzhou, Jiangsu, China; University of Pittsburgh, United States of America

## Abstract

Regulatory T cell (Treg)-mediated immunosuppression represents one of the crucial tumor immune evasion mechanisms and is a main obstacle for successful tumor immunotherapy. Hypoxia, a common feature of solid tumors, has been associated with potentiated immunosuppression, decreased therapeutic response, malignant progression and local invasion. Unfortunately, the link between hypoxia and Treg-mediated immune tolerance in gastric cancer remains poorly understood. In our study, Tregs and hypoxia inducible factor-1α were found to be positively correlated with each other and were increased with the tumor progression. A subsequent *in vitro* study indicated that supernatants derived from gastric cancer cells under hypoxic condition, could induce the expression of Foxp3 via TGF-β1. These findings confirmed the crucial role of Tregs as a therapeutic target in gastric cancer therapy and provided helpful thoughts for the design of immunotherapy for gastric cancer in the future.

## Introduction

Gastric cancer represents one of the most common causes of cancer-related deaths worldwide [1]. Despite significant advances in diagnostic and therapeutic approaches during the last decades, the prognosis of gastric cancer remains dismal because of its high recurrence and metastasis [2]. Immunocytes have long been recognized as a factor promoting tumor growth in the tumor microenvironment [3], however, the underlying molecular basis remains largely enigmatic. A better understanding of the mechanisms in gastric cancer will be beneficial to develop effective therapeutic schemes.

Intratumor hypoxia is a common feature of solid tumors, which might influence the progression of tumors by activating key biochemical and cellular pathways [4], [5], [6]. Studies also demonstrated that hypoxia plays a pivotal role in tumor-mediated immune suppression, contributing to maintain the immunological escape of tumors [7], [8]. Recently, a confirmed link between tumor hypoxia and immune tolerance through the recruitment of regulatory T cells (Tregs) has been established in ovarian cancer [9]. Thus, a hypothesis has been presented that hypoxia may provide obstacles for therapeutic immune interventions.

Increased Tregs have been reported in the circulation and tumor tissues of patients with various cancers, including gastric cancer, colorectal cancer, hepatocellular carcinoma and pancreatic cancer [10]. Accumulating data also indicated that the presence of Tregs resulted in an increased risk for the progression of cancer [11], [12], [13]. Moreover, Treg-mediated immunosuppression is considered to be one of the crucial immune evasion mechanisms in tumor whereby they are able to overcome the anti-tumor activity of CD8 cytotoxic cell, dendritic cell and natural killer cell [14]. Thus, elucidation of the underlying mechanism of Treg enrichment in gastric cancer will be of value for targeting Tregs for a beneficial clinical outcome. Although the mechanisms are not well understood, some studies have indicated that TGF-β1 is involved in it [15].

In this study, we hypothesized that gastric cancer may acquire a selective advantage by induction of Tregs under hypoxia via TGF-β1 signaling pathway thereby evading immune surveillance. To demonstrate our hypothesis, we analyzed the expression of hypoxia inducible factor-1α (HIF-1α) and Foxp3 in gastric cancer tissues, assessed the effect of hypoxia on TGF-β1 production in cancer cell lines, and finally elucidated the role of hypoxia mediating Treg enrichment in gastric cancer.

## Materials and Methods

### Patients and Specimens

Paraffin-embedded, formalin-fixed tumor sections were obtained from 99 patients with gastric cancer that underwent surgical resection from December 2006 to February 2008 at the Second Clinical Medical School of Yangzhou University. Follow-up data were summarized as of October of 2012 with a median follow-up of 53 months (range 4–70 months). Overall survival (OS) was defined as from the time of surgery to last follow-up or time of death.

Peripheral blood samples were collected from 20 patients with gastric cancer one day before and 10 days after surgery from July 2012 to October 2012. Sera were frozen at −80°C immediately after centrifugation for future use. Peripheral blood mononuclear cells (PBMCs) were isolated immediately by a Ficoll density gradient.

None of the patients had received immunosuppressive drugs or chemotherapy before surgical resection. The study was approved by the Ethics Committee (Ethics Committee of Second Clinical Medical School of Yangzhou University, Yangzhou), and written informed consents were obtained from all study participants.

### Immunohistochemical Staining

Serial sections (5 µm) of formalin-fixed, paraffin-embedded specimens were made. Sections were deparaffinized and rehydrated in graded alcohol. Antigen retrieval was performed by treating the slides in EDTA buffer in a microwave for 10 minutes. Rabbit anti-human HIF-1α primary antibody (bioworld, USA), mouse anti-human TGF-β1 antibody (Santa Cruz, USA) and mouse anti-human Foxp3 antibody (Abcam, USA) were used for the primary antibodies. Diaminobenzidine (DAB) was used for substrate following counterstaining with hematoxylin for single staining. Double staining was performed with 2 different chromogens: DAB chromogen (brown color) for HIF-1α and fast-red chromogen (red color) for TGF-β1 or Foxp3.

### Immunoreactivity Scoring

HIF-1α nucleus staining was evaluated following the reported method [16], staining score = intensity of immunoreactivity (IR) × proportion of positively stained cells. IR intensity was stratified into four categories: 0, no IR; 1, weak IR; 2, moderately strong IR; and 3, strong IR. The proportion of positive cells was classified into five groups: 0, no staining; 1, <2% staining; 2, 2–10% staining; 3, 11–29% staining; and 4, >30% staining.

A modified scoring system was used for Foxp3^+^ cells [17]. Ten fields were counted at high-power for Foxp3. The absolute number of lymphocytes per high-power field (HPF 400) was determined. The mean number of positive stained cells per high-power fields was calculated.

### Cell Culture and Hypoxia Induction

Human gastric cancer cell lines, AGS and BGC823, were obtained from the Shanghai Institute of Cell Biology, Chinese Academy of Sciences (Shanghai, China). All cells were propagated in 5% CO_2_ at 37°C in DMEM or RPMI 1640 (HyClone Tianjin, China) supplemented with 10% FBS (Gibco, Australia), 100 U/ml penicillin and 100 mg/ml streptomycin (HyClone Tianjin, China). For hypoxia experiments, cells were seeded into 6-well plates, washed twice with PBS when they grew to 60–80% confluence, kept in serum-free AIMV medium (Invitrogen, USA) for an incubation overnight, and then placed into a modular incubator chamber (Ruskinn Works, YK) for 24 h under hypoxic (1.0% O_2_) or normoxic (21% O_2_) conditions, both with 5% CO_2_ at 37°C. Supernatants were collected and stored at −80°C for later determination of concentrations of TGF-β1 and for co-culture assay. All *in vitro* validation experiments were carried out at least triplicate.

### Immunofluorescence

The cells (1×10^5^/well) were plated onto coverslips in a 24-well plate for a 24 h culture. Subsequently, they were fixed in cold methanol for 30 min to be permeabilized with 0.5% TritonX-100 for another 30 min. Cells were washed thrice, blocked by 10% goat serum in PBS for 30 min, and incubated with anti-human TGF-β1 antibody (Santa Cruz, USA) and anti-human HIF-1α antibody (Epitomics, USA) in a freezer at 4°C overnight. The primary antibody was detected via alexa488-conjugated second antibody (Invitrogen, USA) and Alexa fluor 594-conjugated second antibody (Invitrogen, USA) respectively. The coverslips were examined under OLYMPUS florescence microscopy.

### RNA Extraction and Quantitative Real-time PCR (qPCR)

Each of the two cell lines described above was cultured in triplicate wells under normoxic or hypoxic conditions, as described above. Total RNA was immediately isolated from normoxic or hypoxic cells at the end of the experiment, using Trizol reagent (Invitrogen, USA). Total RNA (5 ng) was reverse transcribed using cDNA Reverse Transcription kits (TaKaRa, Dalian, China), according to the manufacturer’s instructions. qPCR was conducted in a SYBR Green PCR Master Mix (TaKaRa, Dalian, China). Fold changes in the expression of each TGF-β1 mRNA relative to GAPDH were calculated based on the threshold cycle (Ct) as 2^-Δ^ ^(ΔCt)^, where ΔCt = Ct_targe_t - Ct_GAPDH_ and Δ(ΔCt) = ΔCt_hypoxia_ - ΔCt_normoxia._ The TGF-β1 primers were: forward, 5′-GCCAG AGTGG TTATC TTTTG ATG-3′; reverse, 5′- AGTGT GTTAT CCCTG CTGTC AC -3′, and the GAPDH primers were: forward, 5′- GGACC TGACC TGCCG TCTAG AA-3′; reverse, 5′-GGTGT CGCTG TT GAAGT CAGAG-3′. qPCR was performed in triplicate.

### Enzyme-linked Immunosorbent Assay (ELISA)

The concentration of TGF-β1 in the culture supernatants and sera was determined using an ELISA kit (eBioscience, San Diego, CA) according to the manufacturer’s instructions.

### Isolation and Culture of T cells

PBMCs from donors were indirectly labeled with biotin-antibody bocktail and anti-biotin MicroBeads. CD4^+^ T cells were separated by negative selection according to the manufacturer’s instructions (Miltenyi Biotec, Germany). CD4^+^ T cells were directly labeled with CD25 Micro-Beads and CD4^+^CD25^-^ T cells were isolated by negative selection according to the manufacturer’s instructions (purity >95%; Miltenyi Biotec, Germany). The supernatants collected from cultured AGS cells under normoxic or hypoxic conditions were diluted with fresh AIMV medium (1∶3), called normoxic medium and hypoxic medium, respectively. A total of 1.0×10^6^/ml CD4^+^CD25^-^ T cells were stimulated with anti-CD3/CD28-coated beads (1∶5; Invitrogen, USA) ± IL-2 (200 U/ml; Peprotech) for 72 h in AIM-V serum-free medium (control medium), normoxic medium or hypoxic medium. In some experiments, recombinant human TGF-β1 (2 ng/ml; R&D Systems, USA) and TGF-β1 receptor kinase inhibitor LY-364947 (5 µM; Merck, Germany) were added to the cultures.

### Flow Cytometry

Phenotype analysis of Tregs was performed with a BD FACS AriaII flow cytometer (BD, USA). Briefly, the cells were labeled with CD4-FITC and Foxp3-PE (eBioscience, San Diego, CA) following the manufacturer’s protocol. To analyze the prevalence of Tregs, CD4^+^Foxp3^+^ T cells were evaluated after gating on CD4^+^ T cells and were expressed as a percentage of the total CD4^+^ T cells.

### Statistical Analysis

Statistical analyses were conducted on SPSS 17.0 for Windows. Differences between groups were analyzed using a one-way ANOVA, Mann-Whitney U test or χ^2^ test, where appropriate. The correlation was examined by a Pearson correlation. Cumulative survival time was calculated via Kaplan-Meier and analyzed via the log-rank test. Data are expressed as means ± SEM. Statistical significance was set at *P*<0.05.

## Results

### HIF-1α and Foxp3 Expression in Gastric Cancer Tissues

**Figure 1:**
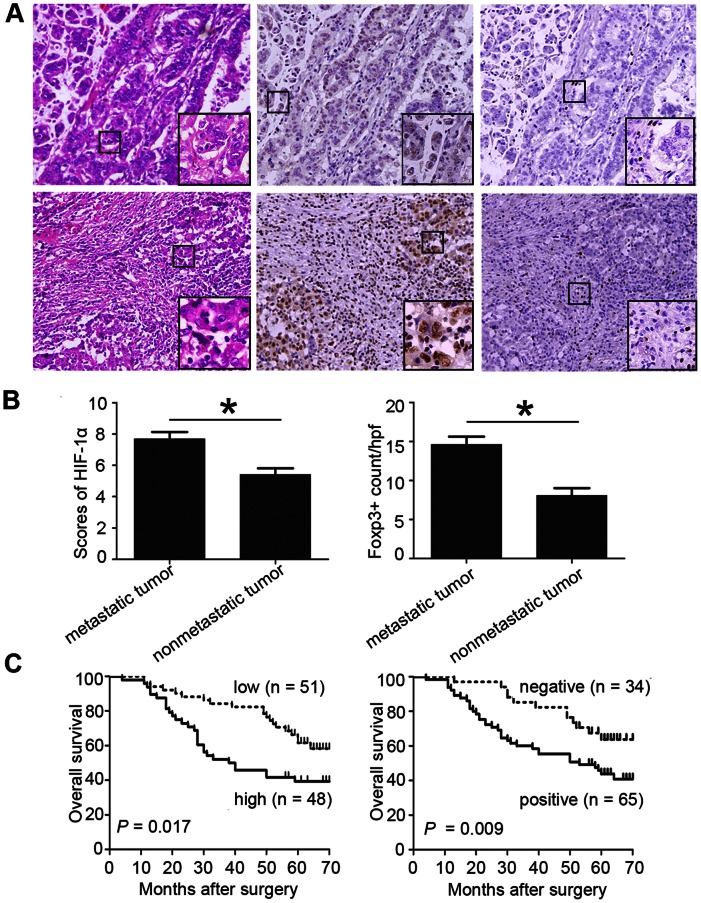
HIF-1α and Foxp3 expression in gastric cancer specimens. (A) Foxp3 and HIF-1α expression in gastric cancer specimens (serial sections) were determined by H&E and immunohistochemistry staining. Representative H&E staining (upper-left and down-left) and images from tumors expressing small (upper and upper-right) or large (down and down-right) amounts of HIF-1α and Foxp3, respectively. (B) Histograms showed that the expression of HIF-1α (left) or Foxp3 (right) are significantly higher in metastatic tumor than in nonmetastatic tumor. (C) Kaplan-Meier analysis was performed to compare overall survival between patients with high and low expression of Foxp3 (left) as well as positive and negative expression of HIF-1α (right). Original magnifications: ×100; ×400 (insets); Values represent mean ± SEM. *P<0.001.

After histological confirmation of gastric cancer (Fig. 1A, upper-left and down-left), we investigated the expression of HIF-1α and Foxp3 by immunohistochemistry in 99 patients. HIF-1α with low expression (Fig. 1A, upper) and high expression (Fig. 1A, down) was found predominantly in the cytoplasm and nucleus of cancer cells at the tumor margin, and only nuclear HIF-1α staining was scored. It is interesting to note that, in some cases, lymphocytes also showed strong expression of HIF-1α. Foxp3 with low expression (Fig. 1A, upper-right) and high expression (Fig. 1A, down-right) was seen in the nucleus of lymphocytes, most of which were located at the tumor margin.

Next, the expressions of HIF-1α and Foxp3 in tumor tissues were compared in patients with nonmetastatic (n = 52) and metastatic (n = 47) gastric cancer. Both the average score of HIF-1α and the number of Foxp3^+^ cells in metastatic patients was higher than in nonmetastatic patients (HIF-1α, 7.69±0.45 vs 5.41±0.40, *P*<0.001, Fig. 1B, left; Foxp3, 14.61±1.01 vs 8.07±0.95, *P*<0.001, Fig. 1B, right).

### Relationship between OS and HIF-1α or Foxp3 in Gastric Cancer Tissues

As of last follow-up, 50 of 99 (50.5%) patients were alive, with a median follow-up of 64 months (range 57–70), whereas 49 had died of disease, with a median time to death of 28 months (range 4–64). To elucidate the relationship between the expression of HIF-1α or Foxp3 and OS, HIF-1α was categorized as negative (n = 34; scores ≤4) or positive (n = 65; scores >4), while Foxp3 was categorized as low (n = 51F) or high (n = 48) using the median number (8.6 cells/HPF) as the cutoff. In our study, OS in Foxp3-low group was significantly higher than in Foxp3-high group (*P* = 0.017, Fig. 1C, left), while HIF-1α-negative group was significantly higher than the HIF-1α-positive group (*P* = 0.009, Fig.1C, right).

### Association between HIF-1α or Foxp3 and Clinicopathologic Characteristics

The correlation between clinicopathological features and expression of HIF-1α or Foxp3 was subsequently evaluated (Table 1). No correlation was found between HIF-1α or Foxp3 expression and age or gender of patients, as well as tumor size. However, there was an increase in the positive rate of HIF-1α with depth of tumor invasion (*P = *0.013). A similar trend was also found between the expression of HIF-1α (*P*<0.001) or Foxp3 (*P* = 0.008) and tumor stage. Additionally, a significant correlation was found between lymph node metastasis and the expression of HIF-1α (*P* = 0.029) or Foxp3 (*P*<0.001).

**Table 1:** Clinicopathological factors associated with Foxp3 or HIF-1α expression.

Charactertistics	HIF-1α		*P*	Foxp3		*P*
	Negative	Positive		Low	High	
No. of patients	34	65		51	48	
Age, ≤60/>60 years	13/21	36/29	0.105	26/25	23/25	0.761
Sex, Male/Femal	20/14	50/15	0.060	33/18	37/11	0.176
Tumor size,≤5/>5 cm	20/14	40/25	0.793	33/18	27/21	0.389
Tumor position,proximal/distal	4/30	7/58	0.881	6/45	5/43	0.831
Depth of invasion,T1+T2/T3+T4	22/12	25/40	0.013	26/25	21/27	0.472
Lymph nodemetastasis, No//Yes	23/11	29/36	0.029	39/12	13/35	<0.001
Stage, I+ II/III+IV	32/2	40/25	<0.001	43/8	29/19	0.008

### Relationship between HIF-1α and Foxp3 or TGF-β1 in Gastric Cancer Tissues

**Figure 2:**
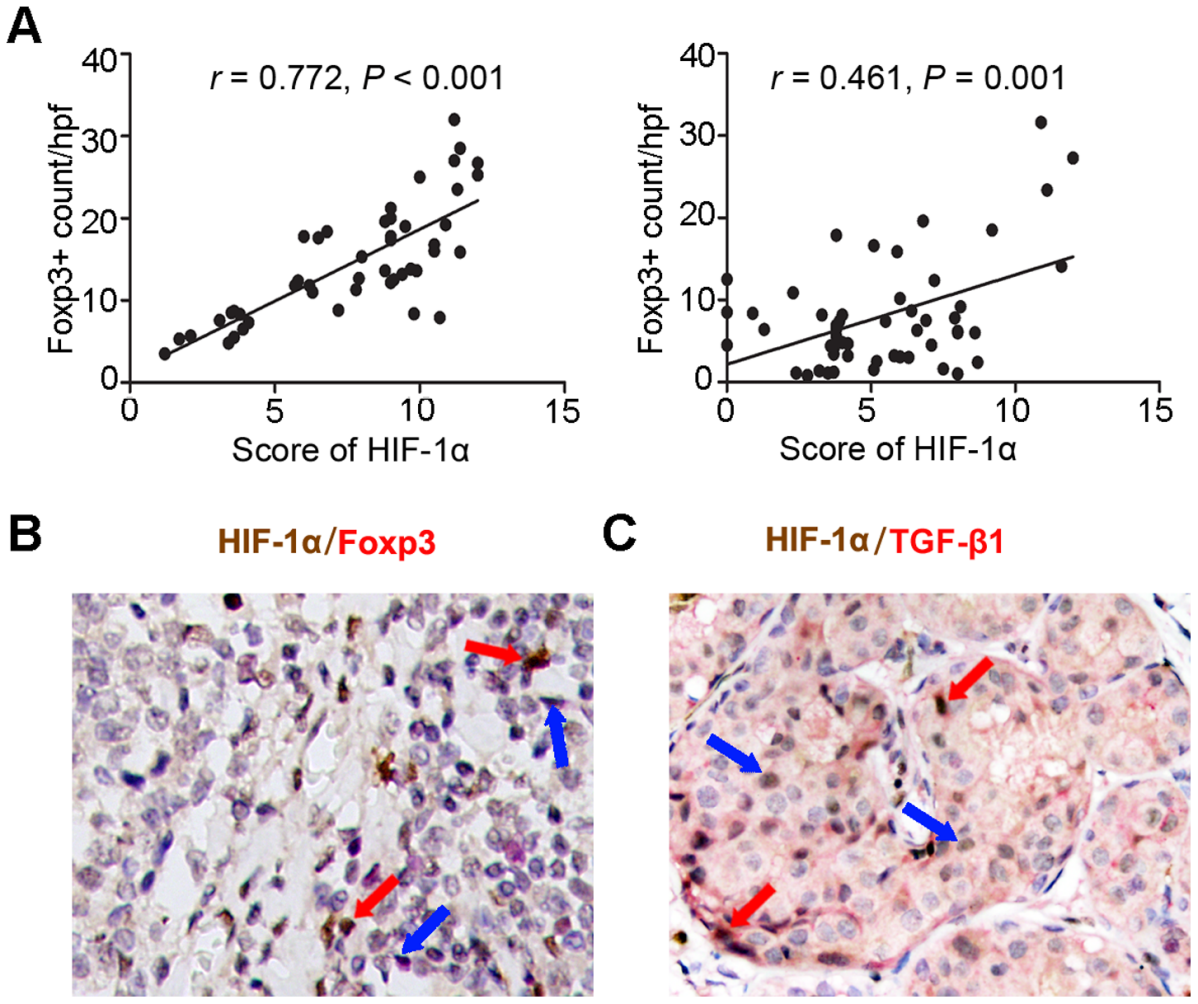
Relationship of HIF-1α and Foxp3 or TGF-β1 in gastric cancer. (A) The correlation of HIF-1α and Foxp3 in metastatic (left) and nonmetastatic tumor (right), as determined by using immunohistochemistry staining. (B) Double staining of HIF-1α (brown, nucleus of cancer cells) and Foxp3 (red, nucleus of lymphocytes). Red arrows indicated tumor cells expressing high HIF-1α, and blue arrows Foxp3^+^ cells. (C) Double staining of HIF-1α (brown, nucleus of cancer cells) and TGF-β1 (red, cytoplasm of cancer cells). Red arrows indicated tumor cells co-expressing high HIF-1α and TGF-β1, and blue arrows for high HIF-1α. Original magnification ×200.

The above-mentioned immunohistochemical result indicated that the number of Foxp3^+^ cells was of strong positive correlation with the score of HIF-1α in patients with metastasis *(r* = 0.772, *P*<0.001; Fig. 2A, left), but not in patients without metastasis (*r = *0.461, *P* = 0.001; Fig. 2A, right). We then performed immunohistochemical double-staining to evaluate the presence of coexpression between HIF-1α and Foxp3 or TGF-β1. The results showed that Foxp3^+^ cells mainly assemble in the sites close to hypoxic tumor region with high HIF-1α expression (Fig. 2B). Furthermore, most high HIF-1α tumor cells were high expression of TGF-β1 (Fig. 2C).

### Circulating Tregs and TGF-β1 in Pre-surgery and Post-surgery Patients

**Figure 3:**
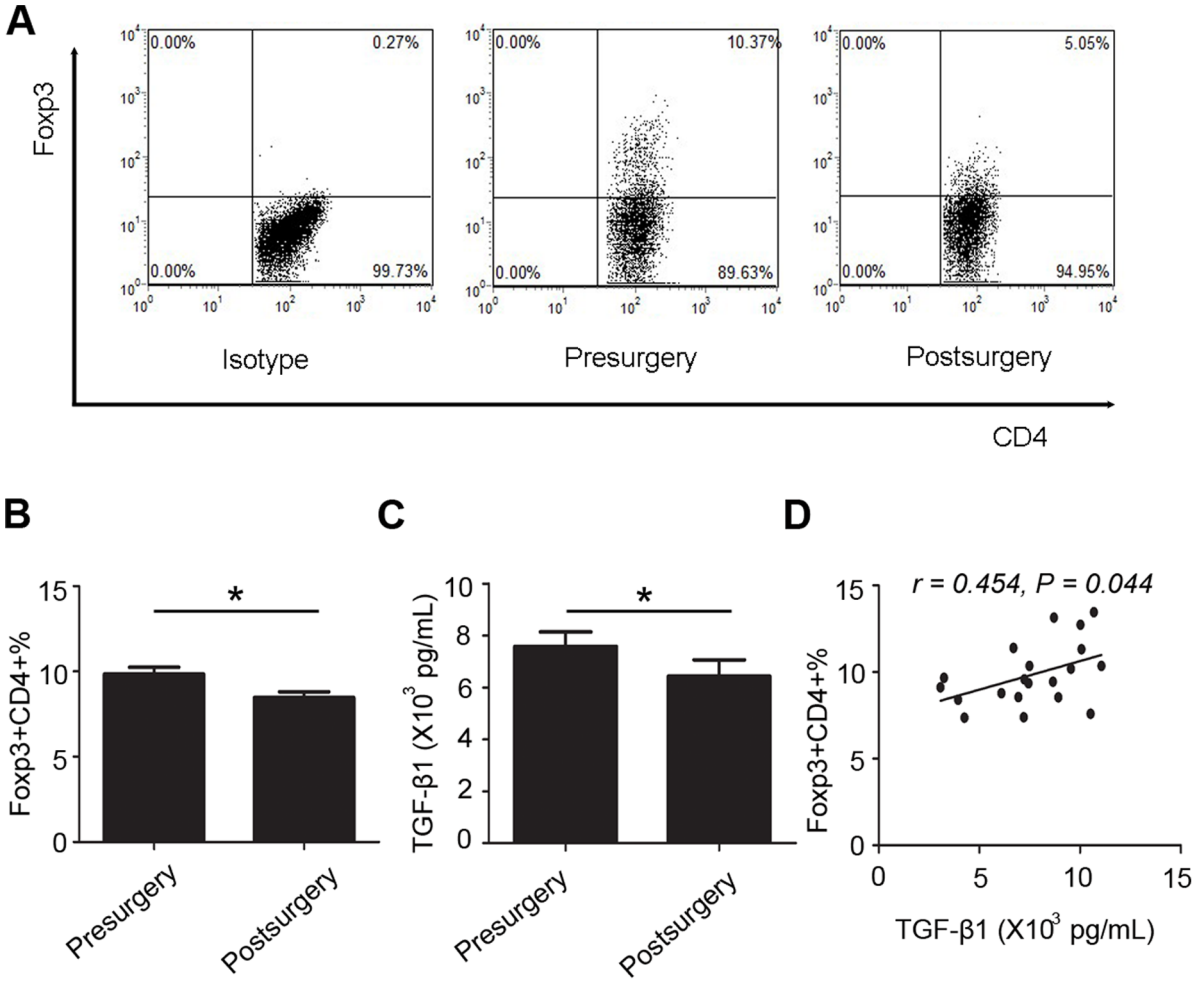
Tregs frequency and TGF-β1 concentration in peripheral blood of 20 gastric cancer patients. (A) Representative plots showed the frequency of CD4^+^Foxp3^+^ T cells before and 10 days after surgery in the peripheral blood of gastric cancer patient, as determined by flow cytometric analysis. (B) A histogram showed the frequency of CD4^+^Foxp3^+^ T cells in peripheral blood of gastric cancer patients before and 10 days after surgery. (C) A histogram showed the concentration of TGF-β1 in peripheral blood of gastric cancer patients before and 10 days after surgery. (D) Correlation of the CD4^+^Foxp3^+^ T cells frequency and the TGF-β1 concentration in peripheral blood of 20 gastric cancer patients before surgery. Values represent mean ± SEM. *P<0.001.

To elucidate the relationship between Tregs and the concentration of TGF-β1 in the serum, CD4^+^Foxp3^+^ T cells were analyzed in patients with gastric cancer via flow cytometry (Fig. 3A). The frequency of Tregs before surgery was higher than that after surgery (9.84±0.40% vs 8.47±0.33%, *P*<0.001, Fig. 3B). TGF-β1 concentrations after surgery decreased significantly when compared with those before surgery (6431.95±629.24 vs 7581.56±556.45 pg/ml, *P* = 0.005, Fig. 3C). A positive correlation between Treg frequency and TGF-β1 concentration was found in serum before operation (*r* = 0.454, *P* = 0.044, Fig. 3D).

### TGF-β1 is Highly Upregulated in Gastric Cancer Cell Lines under Hypoxia

**Figure 4:**
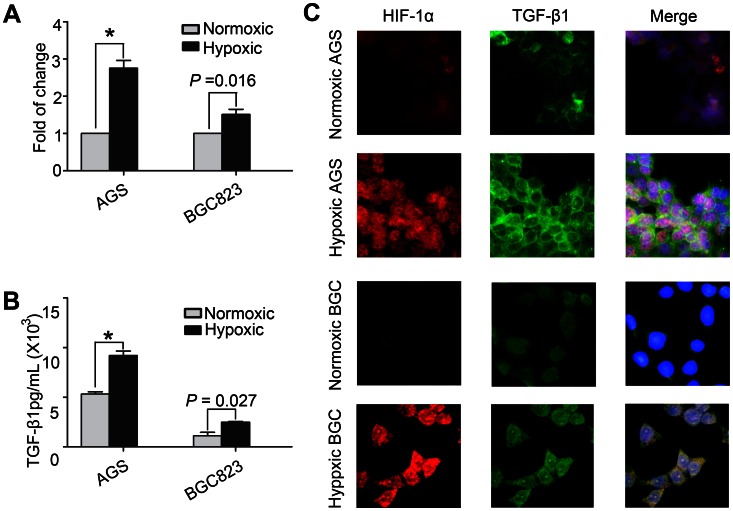
The expression of TGF-β1 in gastric cancer cell lines under hypoxic or normoxic conditions for 24 h . (**A**) A histogram showed the mRNA expression of TGF-β1 in AGS and BGC823 cell lines. **(B**) A histogram showed TGF-β1 level in the culture supernatant of the two cell lines. (**C**) Immunofluorescent double staining showed TGF-β1 and HIF-1α expression in the two cell lines. Alexa fluor594 (nuclear red), alexa488 (indocarbocyanin green) and DAPI (nuclear–counterstaining blue). Original magnification × 200. Values represent mean ± SEM. *P<0.001.

To identify whether hypoxia induces the secretion of TGF-β1 in gastric cancer cells, the TGF-β1 levels were investigated in two gastric cancer cell lines, AGS and BGC823, under hypoxic or normoxic conditions. The mRNA expression of TGF-β1 under hypoxia was increased compared with normoxic conditions (Fig. 4A). Consistent with qPCR, the TGF-β1 levels in culture supernatant showed similar results (Fig. 4B). Further, the expressions of TGF-β1 and HIF-1α were also detected by immunofluorescence, and the results showed increased TGF-β1 and HIF-1α expression under hypoxic conditions when compared with that of under normoxic conditions (Fig. 4, C).

### Hypoxia Augments the Ability of Inducing Tregs via TGF-β1 in Gastric Cancer Cell Lines

**Figure 5:**
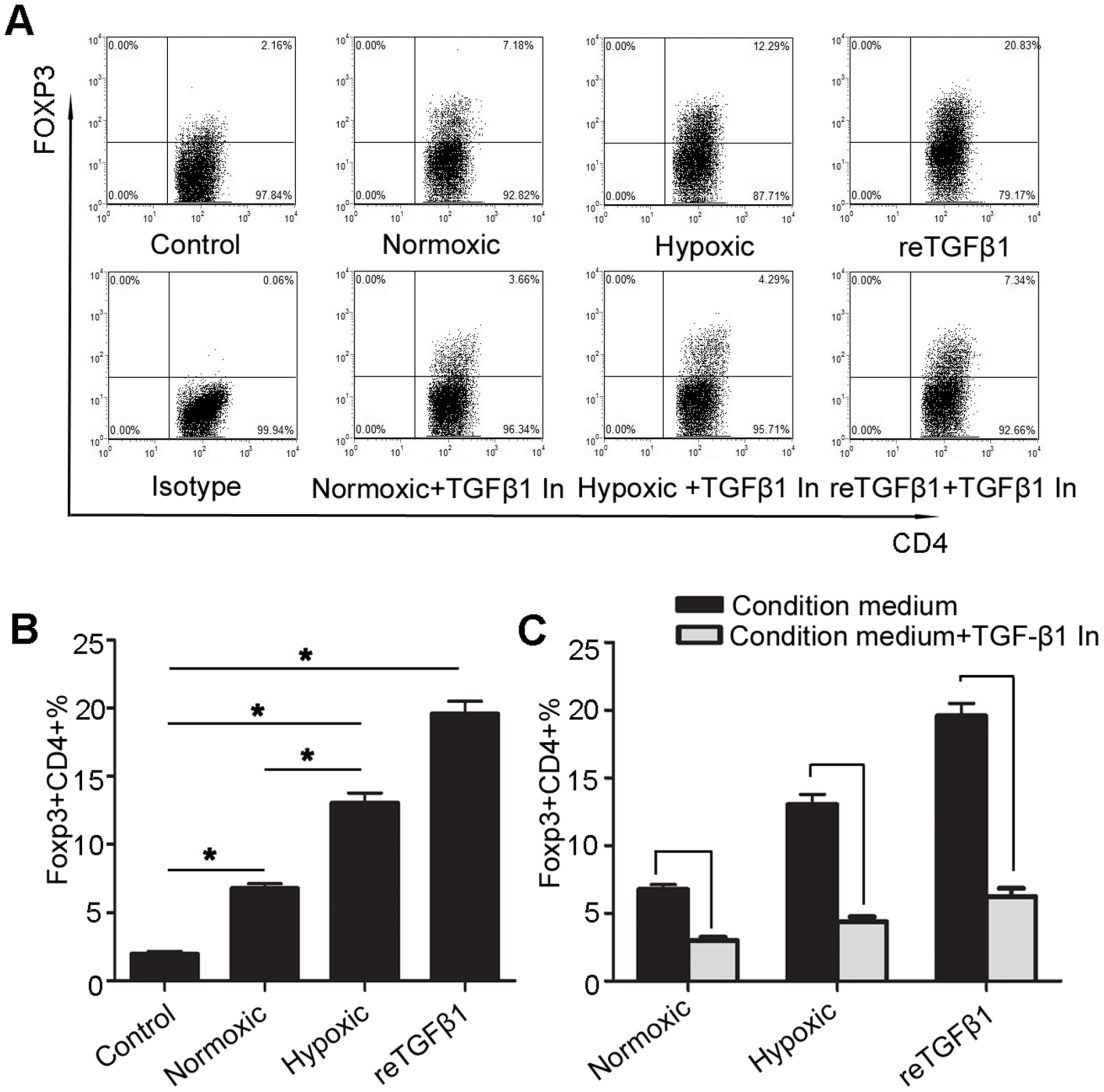
Hypoxia promotes tumor cells to secrete TGF-β1 enhancing Foxp3 expression of T cells. (**A**) Representative plots showed Foxp3 expression in CD4^+^CD25^-^ T cells cultured with different media for 72 h. The supernatants of AGS cells cultured under normoxic or hypoxic condition were called normoxic medium and hypoxic medium, respectively, while AIM-V medium with or without recombinant human TGF-β1 (reTGF-β1) was named reTGF-β1 medium and control medium, respectively. (**B**) A histogram showed Foxp3 expression in CD4^+^CD25^-^ T cells cultured with different media. (**C**) A histogram showed that TGF-β1 receptor kinase inhibitor LY-364947 (TGF-β1 In) partly ablates the indution of Foxp3 in CD4^+^CD25^-^ T cells cultured in different media. Values represent mean ± SEM. * *P*<0.001.

To test the hypothesis that hypoxia-induced TGF-β1 contributes to the increase of Tregs, circulating CD4^+^CD25^-^ T cells from healthy individuals were treated with supernatants from cells under different conditions. The dot plots showed different media inducing the expression of Foxp3 in CD4^+^ T cells (Fig. 5A). The Foxp3 expression of T cells in normoxic medium, hypoxic medium and recombinant human TGF-β1 medium were all increased as compared with the control medium (*P*<0.001). In addition, Foxp3 expression in the hypoxic medium was significantly higher than in the normoxic medium (13.04±0.74% vs 6.76±0.35%, *P*<0.001, Fig. 5B). Further, as LY-364947 was added to each kind of medium, the Foxp3 expression decreased overtly (*P*<0.001, Fig. 5C).

## Discussion

Over the past decade, emerging evidence has suggested that Tregs contribute to the establishment of tumors by impairing the antitumor immune response, or even stimulate metastasis directly [18], [19]. Evidence obtained in recent years demonstrated increased frequencies of intratumoral Tregs in gastric cancer, which have been linked to unfavorable clinicopathological features and poor prognosis [20], [21], [22]. Consistent with these previously reported results, our study provided evidence that the frequency of tumor-infiltrating Tregs increased with advancing tumor stages. Furthermore, Tregs also provided a prognosis factor for post-surgery of gastric cancer. In addition, we confirmed that the numbers of Tregs in metastatic gastric cancer are significantly higher than in nonmetastatic gastric cancer. The above results lend support to the notion that Tregs may contribute to tumor progression and can be a potential therapeutic target for gastric cancer. We thus next attempted to explore the underlying mechanism mediating Treg enrichment in gastric cancer.

Tumor hypoxia is well recognized as a key factor resulting in treatment resistance and poor prognosis for gastric cancer [6], [16], [23]. HIF-1α, as the principal molecular signature for hypoxia, is the main downstream regulator of the hypoxic response in tumor cells [24]. Results from our study demonstrated that HIF-1α correlated with a malignant behavior category, including depth of invasion, lymph node metastasis and advancing tumor stage. Strikingly, gastric cancer patients with high HIF-1α had poorer survival than those with low HIF-1α, which further strengthened our proposal that HIF-1α, or rather hypoxia, is connected with the progression of gastric cancer.

It has long been recognized that tumor cells are protected from immune damage in hypoxic tumor microenvironments; however, the underlying mechanisms still remain under scrutiny [25]. As of now, little is known about whether there is a link between hypoxia and Treg-mediated immunosuppression in tumors. Several recent studies have studied hypoxic regulation of Tregs, with conflicting results. Ben-Shoshan *et al.* [26] reported that hypoxia increases the number and suppressive properties of Tregs to dictate an anti-inflammatory program. Wei *et al.* [7] also confirmed that hypoxia increases the ability of glioblastoma multiforme to induce Tregs, and thus plays a key role in tumor-mediated immune suppression. However, Dang et al. [27] found that HIF-1α inhibits Treg differentiation by targeting Foxp3 protein for degradation. In our study, we presented a strongly positive correlation between Tregs number and HIF-1α expression, suggesting that hypoxia may contribute to Tregs infiltration in gastric cancer. It is worth noting that, in our studies, most Tregs were located in the region close to cancer cells expressing HIF-1α. Thus, further elucidation of whether hypoxia-associated molecules released from cancer cells contribute to the enrichment of Tregs in gastric cancer is needed.

While the mechanisms involved in the enrichment of Tregs in tumors are still not well understood, We and other investigators have recently demonstrated that tumor cells can serve as a source of TGF-β1, which is required for the induction and maintenance of Tregs *in vitro* and *in vivo* [28], [29], [30]. In the current study, the frequency of Tregs and TGF-β1 in circulation before surgery was higher than that after surgery, and the two parameters showed a positive correlation, suggesting that tumor-derived TGF-β1 may contribute to the increase of Tregs in gastric cancer.

Hypoxia, a common feature of cancers, can induce cancer cells to secret immunosuppressive cytokines including TGF-β1 [7], [31]. Results from our study demonstrated most high TGF-β1 cells were cancer cells expressing HIF-1α in tumors. An *in vitro* study showed the expression of TGF-β1 increased to a greater extent in gastric cancer cells under hypoxic culture, which is also consistent with above-mentioned studies. Based on our findings, we hypothesized that cancer cells secrete TGF-β1 to induce the production of Tregs in gastric cancer. To test our hypothesis, isolated CD4^+^CD25^-^ T cells from healthy individuals were cultured in diluted supernatants of tumor cells under different culture conditions. Results showed that Foxp3 expression of T cells cultured in hypoxic medium was significantly higher than cells cultured in normoxic medium. Furthermore, adding extra recombinant human TGF-β1 significantly increased Foxp3 expression, whereas adding LY-364947, a TGF-β1 receptor inhibitor, reduced Foxp3 expression. Thus, these results establish a direct link between hypoxia, TGF-β1 and Tregs in gastric cancer.

However, our preliminary study also showed that TGF-β1 was unaltered by HIF-1α stabilizer, cobalt chloride (CoCl_2_), or HIF-1α inhibitor, 2-methoxyestradiol (2ME2) in AGS and BGC823, although the HIF-1α was increased by hypoxia (data not shown). These findings suggested that HIF-1α may be unassociated with the induction of TGF-β1 in gastric cancer cells under hypoxia, which is consistent with previous study [32]. Furthermore, the production of Tregs could not be completely ablated by blocking TGF-β1, indicating that other cytokines or pathways may also be involved. In addition, cancer cells are not the sole source of cytokines within the tumor microenvironment. Thus, further studies are needed to confirm the underlying mechanism of Treg accumulation in gastric cancer.

In conclusion, the present study demonstrated that hypoxia potentiates the ability of gastric cancer to evade immune surveillance by up-regulating the expression of TGF-β1, thereby inducing Tregs in tumors. Further, our data also provided evidence for the existence of intercellular cross-talk between tumor cells and Tregs, which might regulate anti-tumor immune responses.
